# Addressing social determinants of health in the field of ophthalmology: In response to: Identifying and addressing social determinants of health to improve patient-centered care – Kalsi N, Gordon D, Geske J.

**DOI:** 10.1017/cts.2024.596

**Published:** 2024-09-20

**Authors:** Imani Nwokeji, Chisom Madu, Jin Ming Lin

**Affiliations:** CUNY School of Medicine, New York, NY, USA

In 2004, the World Health Organization highlighted the imperative nature of social determinants that shape the well-being of patients, and the pressing need for medical practitioners to confront these prominent inequities that contribute to deteriorating health outcomes [1,2]. Consequently, I fully concur with the author’s assertion regarding the imperative nature of addressing these social determinants of health (SDH) to mitigate the healthcare disparities that negatively affect our healthcare system. Specifically, Kalsi et al. discuss a clinical improvement initiative focused on identifying and remedying these factors, thereby facilitating a more holistic, patient-centric approach to care[1]. SDH play a crucial role in ophthalmology care, extending far beyond merely identifying underserved communities. These determinants, which include factors such as education level, income, transportation access, and health literacy, directly impact a patient’s ability to access and adhere to eye care regimens. Moving forward, this innovative approach to clinical care should be more widely adopted and implemented throughout ophthalmology, especially in the management of diseases like glaucoma.

The Screening and Interventions for Glaucoma and Eye Health Through Telemedicine (SIGHT) studies provide a model for using social determinants screening and community-based interventions to improve vision care[3]. Strategies include community health workers conducting on-site vision screenings, patient navigators, motivational interviewing to increase adherence to follow-up eye exams, and telehealth methods to facilitate access to eye care for those diagnosed with glaucoma or other eye diseases. Key outcomes measured are visual acuity, patient-reported vision quality of life, detection rates of eye diseases, and adherence to recommended follow-up exams. Patient satisfaction is also evaluated after vision screenings and eye exams across multiple clinical sites[4]. Implementing such an approach in underserved areas like the Bronx in New York City could help identify and address critical social determinants that impact adherence to glaucoma care amongst other ophthalmic conditions[4]. While initially focused on underserved areas, the principles of SIGHT can be adapted for various populations. The program uses SDH screening to tailor interventions to individual patient needs, not just at the community level. For example, identifying transportation barriers through SDH screening can lead to targeted solutions like providing transportation assistance or implementing telemedicine options, thereby improving adherence to glaucoma management plans. This aligns with the broader goal of using standardized social determinants screening tools in a streamlined clinical workflow to identify and provide resources for overcoming barriers to health and well-being in diverse patient populations.

An econometric analysis using a Markov model projected that implementing an AI-assisted glaucoma screening program similar to SIGHT in remote areas of China could lead to healthcare cost savings of $1.2 million over 15 years by preventing progression to advanced disease stages requiring more costly treatments [5]. Additionally, it estimated $300,000 in preserved workforce productivity by preventing vision loss and blindness, allowing the elderly to remain productive members of their communities. These findings demonstrate the economic incentives for ophthalmology to embrace initiatives that identify and address social determinants impacting adherence and equitable access to vision care in underserved populations with a high glaucoma burden[5].

Recent research has underscored the crucial role of ophthalmologists and recognized their significant contribution to delivering comprehensive primary care to patients[6]. Ophthalmologists can detect and diagnose an array of systemic conditions that may have ophthalmic manifestations, even before the onset of symptoms. Numerous studies have demonstrated that routine eye examinations can unveil early indicators of chronic diseases such as diabetes, hypertension, and autoimmune disorders[6]. Thus, early detection serves significant clinical importance and mitigates the progressions of many conditions.

The impact of social determinants on healthcare outcomes is increasingly clear. By recognizing and tackling these disparities, medical professionals – particularly those in ophthalmology – can significantly improve patient health outcomes. And thus prevent the development of glaucoma and subsequently blindness in adversely affected communities. Specifically, the SIGHT and the cost-effectiveness analysis by Xiao *et al*. [5] demonstrate the potential of integrating community-based interventions and telehealth strategies for more equitable access, patient navigation, quality of life, as well as adherence for underserved populations with a high incidence of glaucoma[3]. The economic analysis by Xio *et al*. [5] further highlights the economic benefits of chronic disease management in underserved areas. By executing multifaceted approaches, the field of ophthalmology can steer change in confronting the interplay between social determinants of health and thus promoting holistic and equitable care for patients.
